# Climate Change and Women's Health: A Scoping Review

**DOI:** 10.1029/2021GH000386

**Published:** 2021-09-01

**Authors:** Zalak Desai, Ying Zhang

**Affiliations:** ^1^ School of Public Health University of Sydney Sydney NSW Australia

## Abstract

Climate change is a significant global health threat that is, underpinned by the existing issue of gender inequality. A scoping review was conducted to better understand the relationship between climate change and women's health. We found a notably higher proportion of existing studies focused on low‐ and middle‐income countries (LMICs). Most of the studies included were published after 2010, with predominantly qualitative study designs. Four key themes were identified, including women's exposure to climate change risks, the impacts on women's health, factors contributing to the vulnerability, and responding strategies in addressing climate change. The scoping review indicates that women's health is at higher risks due to the vulnerability to climate change, especially in LMICs. Meanwhile, it is beneficial to have insights from women in terms of adaptation and mitigation strategies to build stronger resilience. Mixed methods are strongly recommended to support evidence‐based policy making in responding to climate change.

## Key Points

- Women's health is at higher risks due to the vulnerability to climate change, especially in low‐ and middle‐income countries (LMICs)
- The societal, cultural, and economic factors contribute to the vulnerability. It is beneficial to have a gender aspect in responses
- Mixed methods incorporating quantitative and qualitative assessments are needed

## 1 Introduction

Climate change is a significant global health issue that has rapidly become a priority on the global health agenda (Duncan, 2006; Levy & Patts, 2015). Its detrimental effects to Earth's ecosystem have led to increases in natural disasters, vector borne diseases, poor air quality and extreme variance in climatic temperatures, all of which directly and indirectly affect human health (Duncan, 2006; Rylander et al., 2013). Abundant research has confirmed the relationship between climate change and human health, highlighting poverty, food insecurity, geographic isolation and degrading societal norms as key factors which exacerbate the negative effect of climate change (Jerneck, 2018; Langer et al., 2015).

Globally, approximately 1.3 billion people in low‐and middle‐income countries (LMICs) live below the poverty line, with 70% of those being female (Sorensen et al., 2018). Climate change exacerbates women's distinct health needs, particularly during pregnancy where maternal health and nutrition is vital to the developing fetus and infant (Franco‐Orozco, 2018; Rao, 2011; Rylander et al., 2013; Sorensen et al., 2018; Watt, 2011). In addition to this, women in LMICs generally have a domestic role in the household, exposing them to poor air quality through inappropriate gases used during cooking and poor ventilation of the cooking area (Bhallamudi & Lingam,, 2019; Duncan, 2006; Mazorra et al., 2020; Pinkerton et al., 2013; Rosenthal et al., 2018; Tirado et al., 2013). In terms of social and cultural issues, women often have less access to ownership of land, education and paid labor, all of which increases their vulnerability to climate change (Jerneck, 2018; Langer et al., 2015). Women are often faced with unequal access to economic and technical resources after natural disasters and climate‐change related extreme weather events (Jerneck, 2018; Langer et al., 2015). There exists a complex relationship between climate change and women's health that is, underpinned by the existing issue of gender inequality (Sorensen et al., 2018; United Nations, 2020; World Health Organization, 2014).

The role of women in tackling climate change in general has been made a priority as part of many recent global agendas, such as the Sustainable Development Goals (SDGs), Paris Agreement on Climate Change and the United Nations Framework Convention on Climate Change, which acknowledge the relationship between climate change and women's health (Collantes, 2018; Haque, 2011; Langer, et al., 2015; Manandhar, et al., 2018; Maurice, 2015; Sorensen et al., 2018; United Nations 2015, 2020). The World Health Organization (WHO) has also highlighted the importance of gender, health and climate change and offered mitigation strategies to address the issues present (World Health Organization, 2014). In addition to these, there has been an increase in the number of published literature that identify this relationship and highlight the need for sustainable solutions to address this issue (Watts et al., 2018). These solutions are based on themes of women empowerment and advocacy for gender equality, through community‐led strategies, national policies and global resilience (Dulal et al., 2009; Engelman, 2010; The Lancet, 2015; Paavola, 2008; Page & Maja, 2010; Sen Roy, 2018).

Despite this issue being identified as an increasing global concern, no single study has been able to identify the breadth of literature available around this topic and explore all aspects of the relationship between climate change and women's health. The study aims to fill in the gap in literature by conducting a scoping review to better understand climate change and women's health to support the development and implementation of climate change strategies and actions.

## 2 Methods

As defined by Arksey and O'Malley, a scoping review aims to map the key concepts that underpin a research topic and highlight main sources and various types of evidence available (Arksey & O'Malley,, 2005). The methodological framework by Arksey and O'Malley was adopted for the review.

A systematic search of literature was undertaken using four databases, including MEDLINE, EMBASE, CINHAL and SCOPUS. Key words and search strategies were developed and are outlined in Table 1. The set search strategy was developed after initial search on each database to identify relevant topics and MeSH terms. The same search strategy was adopted for each of the four databases to identify literature present and exported to EndNote for further analysis. Citation chaining was also utilized to identify further literature that was not indexed in the databases selected.

**Table 1:** Key Words and Search Strategy^a^

Keywords	Terms used
Climate change/variability/extremes	(Climate w/1 change^a^ OR variab^a^ OR extrem^a^) OR “global warming” OR “greenhouse effect.”
Gender/women	Gender OR wom?n OR “wom?n's health” OR female^a^ OR (gender w/1 role^a^ OR perspective^a^ OR perception^a^ OR disparit^a^ OR equalit^a^).
Health	Health^a^ OR “health outcome^a^” OR wellbeing OR wellness OR “quality of life” OR “health effect^a^.”
Maternal health	(Maternal w/1 health OR mortality OR morbidity OR welfare OR wellbeing) OR “maternal health outcome^a^” OR “maternal health impact^a^.”
Mitigation and adaptation	Sustain^a^ OR mitigat^a^ OR adapt^a^.

^a^ The above search strategy is modified for the SCOPUS database.

Database search was conducted between the months of March and May in 2020, with the last search being conducted on 10/05/2020. The studies retrieved from the databases were exported onto EndNote program for further analysis. Duplicates were removed and the initial title and abstract screening was completed by one reviewer (ZD). After this initial screening, the references selected for full text screening were exported onto a Microsoft Excel spreadsheet. The spreadsheet was organized to extract data from each article including the authors, publication year, publication title, location, population demographics, study design, findings, and limitations. Both reviewers (ZD and YZ) independently performed the full text analysis and extracted relevant data. Discrepancies were resolved by discussion amongst the reviewers.

The set inclusion and exclusion criteria aided in selecting relevant studies for the scoping review. Studies were included if the full text was available, in English language, and published before 31/03/2020. Research that focused solely on air pollution and women's health was not included due to abundance evidence on this topic unless the relationship between air pollution and climate change was also discussed. Moreover, although children's health is closely related to maternal health and women's health in general, studies that only focused on children's health were not included as they were beyond the scope of this review.

## 3 Results

**Figure 1:**
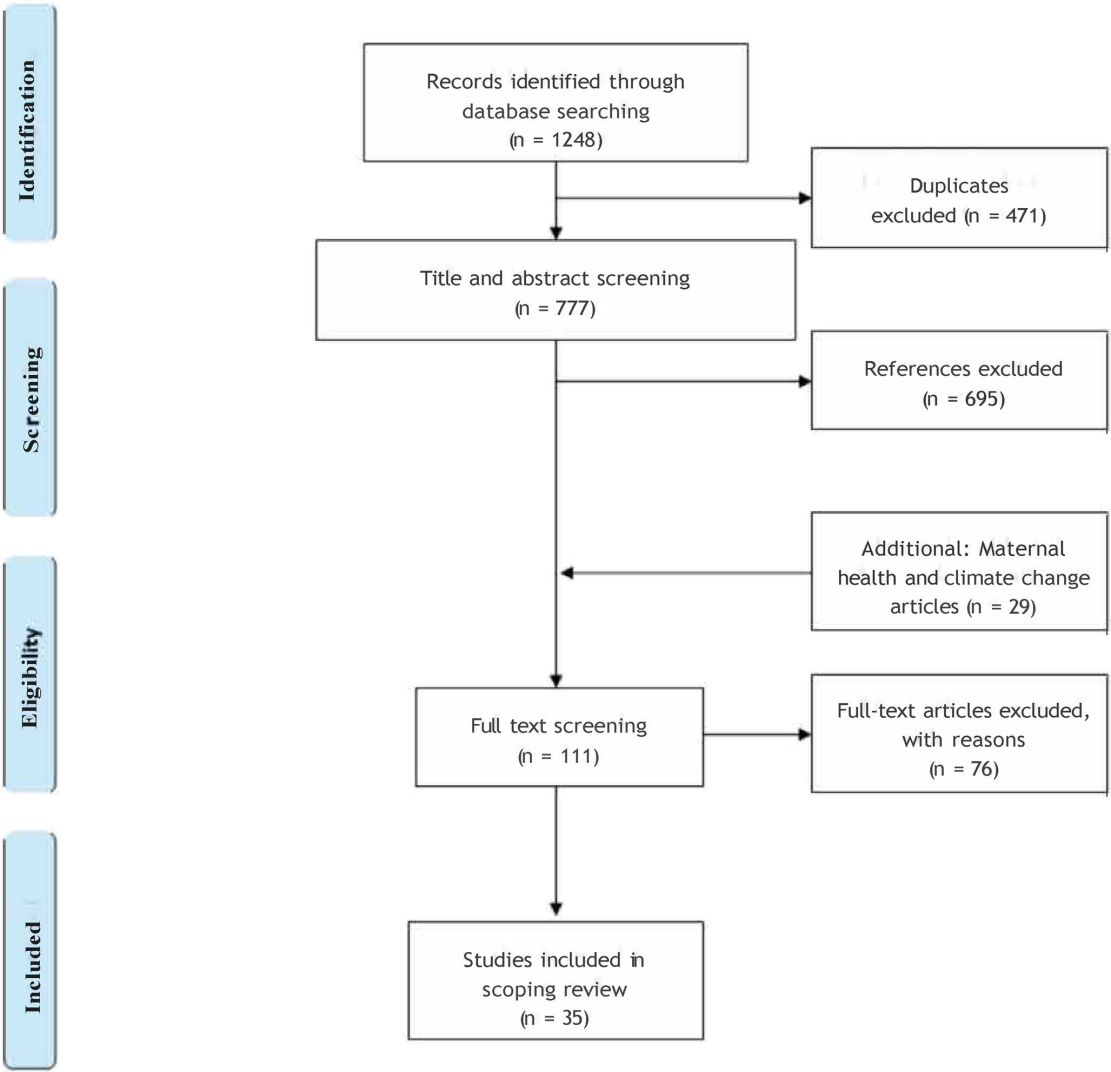
PRISMA flow diagram.

Initial searches on the databases yielded a total of 1,248 citations (see Figure 1), which were exported to EndNote for further analysis. After the removal of duplicates (*n* = 471), title and abstract screening was performed on the remaining unique articles (*n* = 777). The majority of these publications (*n* = 695) were irrelevant to the topic of the scoping review and were excluded at this stage. Full‐text analysis was performed for the remaining publications (*n* = 82). It was during this process that “maternal health” was identified as a relevant topic to “women's health” and search incorporating “maternal health” and “climate change” was performed again on the databases to identify further publications relevant to the topic. After removal of duplicates and title and abstract screening for articles relevant to climate change and maternal health, a total of 29 articles were further identified for full text analysis. Further publications were identified through citation chaining of reference lists and these were again reviewed independently by the two researchers for inclusion in the scoping review. In all, a total of 35 articles were included in the scoping review.

### 3.1 Literature Characteristics

Of all the articles included in the scoping review (*n* = 35), the studies mainly explored the relationship between climate change and women's health in LMICs (*n* = 27). Most of the studies included were published after 2010 (*n* = 32), with only a few being published before this time period (*n* = 3). Most of the articles employed a qualitative study design (*n* = 18). There were a smaller number of quantitative studies (*n* = 11) and even fewer studies which utilized a mixed‐methods study design (*n* = 6). The qualitative study designs obtained responses through individual in‐depth, semi‐structured and structured interviews, focus group discussions, observations, case scenario analyses or a combination of these methods. Quantitative studies utilized cross‐sectional surveys, regression modeling and time‐series study designs to report relevant data. Studies that incorporated a mixed‐methods approach combined a survey or randomized and non‐randomized controlled design with qualitative methods such as use of in‐depth interviews and focus group discussions to further explore research issues. The studies included in the analysis were based in different countries and regions, with notably higher proportion exploring LMICs (*n* = 22). Broadly, the studies focused on topics of climate change exposures and risks, health outcomes, risk factors to vulnerability and mitigation and adaptation strategies that addressed the relationship between climate change and women's health. Most articles had findings across two or more of these themes, however, only three articles were identified to have findings across all four themes (Beaumier & Ford, 2010; Bunce et al., 2016; Denton, 2002). A summary of the articles included in the analysis and their literature characteristics is outlined in Table 2.

**Table 2:** Summary of Literature Included in the Scoping Review

Author, year	Region	Study design	Findings			
			Climate change exposures and risks	Health outcomes	Risk factors to vulnerability	Mitigation or adaptation strategies
Abdullah et al., 2019	Rural Bangladesh	Qualitative	X	X		
Alhassan et al., 2019	Ghana	Mixed methods	X	X	X	
Asamoah et al., 2018	Ghana	Quantitative	X	X		
Balehey et al., 2018	Afar, Ethiopia	Qualitative		X	X	
Beaumier & Ford, 2010	Canada: Igloolik, Nunavut	Qualitative	X	X	X	X
Bunce et al., 2016	Canada: Iqaluit, Nunavut	Qualitative	X	X	X	X
Carranza and Niles, 2019	Kenya, Uganda and Senegal	Qualitative			X	
Cil and Cameron, 2017	Unites States of America	Quantitative	X	X		
Denton, 2002	Global	Quantitative	X	X	X	X
Drolet, 2012	British Columbia, Canada	Mixed methods	X			X
Granderson, 2017	Tongoa Island, Vanuatu	Mixed methods				X
Khan et al., 2011	Bangladesh	Mixed methods	X	X		
Khapung, 2016	Western Nepal	Qualitative		X	X	X
Koehler, 2016	Global	Qualitative		X	X	X
Larson et al., 2018	Brazil, Cameroon, Indonesia, Peru, Tanzania and Vietnam	Quantitative			X	X
Leipert and Reutter, 2005	Northern British Columbia, Canada	Qualitative	X		X	X
MacVicar et al., 2017	Uganda	Qualitative	X	X	X	
Marí‐Dell'Olmo et al., 2019	Barcelona	Quantitative	X		X	
Mason and Agan, 2015	Baguio City, Philippines	Quantitative	X		X	X
Masson et al., 2019	Chad	Quantitative			X	X
Mazorra et al., 2020	Casamance Natural Subregion, West Africa	Qualitative		X	X	X
McCall et al., 2019	Leipzig, Germany	Quantitative	X			X
Ortega‐Egea et al., 2014	Europe	Mixed methods			X	X
Patrick and Teresa, 2011	Victoria, Australia	Qualitative				X
Poudel et al., 2020	Lamjung district, Nepal	Qualitative	X	X	X	
Powers, 2012	Australia	Quantitative				X
Roy and Venema, 2002	India	Qualitative			X	X
Sanchez et al., 2012	Benin, West Africa	Qualitative	X			
Seidel and Bell 2014	Global	Qualitative			X	X
Shanthi et al., 2017	Tamil Nadu, India	Qualitative			X	X
Scheelbeek et al., 2016	Coastal Bangladesh	Quantitative	X	X		
Shodieva et al., 2014	Uzbekistan	Qualitative			X	X
Singh et al., 2018	Karnataka, South India	Mixed methods	X		X	X
Tirado et al., 2013	Nigeria	Qualitative	X	X		X
Zhang et al., 2018	Australia	Quantitative	X			

*Note*. ‘X' indicates that the finding was observed in the article.

### 3.2 Findings on the Four Themes

#### 3.2.1 Women's Exposures to Climate Change Risks

Increase in climate‐related extreme weather events, such as floods, hurricanes, heat waves, droughts, poor air quality and salinity of water, were reported by 20 articles in relation to women's health (Abdullah et al., 2019; Alhassan et al., 2019; Amoroso, 2018; Asamoah et al., 2018; Beaumier & Ford, 2010; Bunce et al., 2016; Cil & Cameron, 2017; Denton, 2002; Drolet, 2012; Khan et al., 2011; Leipert & Reutter, 2005; MacVicar et al., 2017; Marí‐Dell'Olmo et al., 2019; Mason & Agan, 2015; McCall et al., 2019; Poudel et al., 2020; Sanchez et al., 2012; Scheelbeek et al., 2016; Singh et al., 2018; Tirado et al., 2013; Zhang et al., 2018). Floods, hurricanes, heat waves and droughts were found to impact the agricultural industry where women worked as primary labourers, retrieved food for daily consumption and relied upon heavily for household incomes (Alhassan et al., 2019; Denton, 2002; Drolet, 2012; Poudel et al., 2020). Women were found to be more affected by temperature extremes such as heat waves which put them at a higher risk of poor maternal health, hypertension and heat exhaustion (Asamoah et al., 2018; Cil & Cameron, 2017; MacVicar et al., 2017; Marí‐Dell'Olmo et al., 2019; McCall et al., 2019; Singh et al., 2018). Climate change increases the frequency and intensity of extreme weather events, including heavy snowfall and blizzards in the most northern parts of the world, which affected women's ability to find and collect food for their family, as part of their primary caretaker roles in the communities (Beaumier & Ford, 2010; Bunce et al., 2016; Leipert & Reutter, 2005). Melting of ice glaciers due to climate change decreased seafood available in the northern regions, which resulted in food insecurity for women in those communities (Bunce et al., 2016). The rise in sea‐level due to climate change has also increased salinity of water in surrounding sources as rising sea water push saltwater farther upstream, whereby some communities collected salinated water and have been found to cause more maternal health problems (Khan et al., 2011; Scheelbeek et al., 2016). Only one identified study reported that there was no gender difference found in terms of perceptions of climate change (Sanchez et al., 2012). However, the result should be interpreted cautiously because the study did not consider social, cultural and religion factors that could affect how women express opinions. Another Australian study that examined suicide as a health outcome of climate change has found that increasing temperatures are more likely to have a positive correlation with elevated suicide rate among male than female (Zhang et al., 2018).

#### 3.2.2 Impacts on Women's Health

The relationship between climate change and women's health outcomes was analyzed by 16 studies included in the review (Abdullah et al., 2019; Alhassan et al., 2019; Asamoah et al., 2018; Balehey et al., 2018; Beaumier & Ford, 2010; Bunce et al., 2016; Cil & Cameron, 2017; Denton, 2002; Khan et al., 2011; Khapung, 2016; Koehler, 2016; MacVicar et al., 2017; Mazorra et al., 2020; Poudel et al., 2020; Scheelbeek et al., 2016; Tirado et al., 2013). Women were more affected by nutritional deficiencies, such as malnutrition and anemia, due to food insecurity reasons (Beaumier & Ford, 2010; Denton, 2002; Koehler, 2016; Tirado et al., 2013). This was found to be more common in female‐headed households compared to male‐headed households (Alhassan et al., 2019). Women in rural areas were also more likely to be at risk of vector‐borne diseases because they are likely to be in close proximity to wells, rivers and ponds when they collect water supplies (Bunce et al., 2016; Denton, 2002; Poudel et al., 2020). A strong relationship was also identified between climate change and maternal health (Abdullah, et al., 2019; Asamoah et al., 2018; Cil & Cameron, 2017; Denton, 2002; Khan et al., 2011; Khapung, 2016; Koehler, 2016; MacVicar et al., 2017; Scheelbeek et al., 2016; Tirado et al., 2013). Pregnant women were more likely to experience hypertension, exhaustion, miscarriages and stillbirths with higher temperatures and food insecurity (Asamoah et al., 2018; Cil & Cameron, 2017; Khan et al., 2011; MacVicar et al., 2017; Scheelbeek et al., 2016; Tirado et al., 2013). This was more common in women who worked as manual labourers in the agricultural industry (Abdullah et al., 2019; MacVicar et al., 2017). Women developed more respiratory conditions, particularly in rural areas where renewable energy was not available, and women used hazardous traditional biomass to cook foods leading to inhalation of toxic pollutants (Mazorra et al., 2020).

#### 3.2.3 Factors Contributing to the Vulnerability

Twenty‐two articles explored the risk factors to vulnerability in relation to women's health and climate change (Alhassan et al., 2019; Balehey et al., 2018; Beaumier & Ford, 2010; Bunce et al., 2016; Carranza & Niles, 2019; Denton, 2002; Khapung, 2016; Koehler, 2016; Larson et al., 2018; Leipert & Reutter, 2005; MacVicar et al., 2017; Marí‐Dell'Olmo et al., 2019; Mason & Agan, 2015; Masson et al., 2019; Mazorra et al., 2020; Ortega‐Egea et al., 2014; Poudel et al., 2020; Roy & Venema, 2002; Seidel & Bell 2014; Shanthi et al., 2017; Shodieva et al., 2014; Singh et al., 2018). Climate change exacerbated existing gender and social inequalities faced by women, especially in rural and remote communities (Alhassan et al., 2019; Balehey et al., 2018; Beaumier & Ford, 2010; Bunce et al., 2016; Khapung, 2016). Women in rural areas were found to have decreased social networking and employment opportunities in order to increase their income (Alhassan et al., 2019; Beaumier & Ford, 2010; Khapung, 2016; Leipert & Reutter, 2005; Mason & Agan, 2015; Masson et al., 2019; Poudel et al., 2020). In very remote areas, patriarchal nature of the communities enhanced gender discrimination and violence against women when natural disasters destroyed agricultural crops and decreased household income (Leipert & Reutter, 2005; Masson et al., 2019; Roy & Venema, 2002). They were identified as often being the last members to eat in the household, allowing the males in the family and the children to eat first (Bunce et al., 2016; Leipert & Reutter, 2005; Masson et al., 2019; Ortega‐Egea et al., 2014). The studies overall reported that women in general had very limited rights in owning land, wealth and were often excluded from inheritance (Balehey et al., 2018; Beaumier & Ford, 2010; Carranza & Niles, 2019; Denton, 2002; Koehler, 2016; Leipert & Reutter, 2005; MacVicar et al., 2017; Mason & Agan, 2015; Masson et al., 2019; Roy & Venema, 2002; Shanthi et al., 2017; Shodieva et al., 2014; Singh et al., 2018). Women's health and their role as caregivers are significantly affected by their lack of human rights, exclusion from decision making in society, and financial dependence on males who earn income in their households (Masson et al., 2019; Ortega‐Egea et al., 2014; Poudel et al., 2020; Roy & Venema, 2002; Singh et al., 2018). Accessing education is considered a superior privilege for women in rural communities, who are not given opportunities to build careers which may enable them to improve their current socio‐economic status (Beaumier & Ford, 2010; Larson et al., 2018; Marí‐Dell'Olmo et al., 2019; Seidel & Bell 2014; Shanthi et al., 2017; Shodieva et al., 2014). Together, these factors prevent women from accessing opportunities to alleviate their exposures to climate change impacts, thus contributing to the gender‐driven vulnerability to climate change.

#### 3.2.4 Responding Strategies

Twenty‐two articles included in the review discussed mitigation and adaptation strategies to address the negative effects of climate change on women's health (Beaumier & Ford, 2010; Bunce et al., 2016; Denton, 2002; Drolet, 2012; Granderson, 2017; Khapung, 2016; Koehler, 2016; Larson et al., 2018; Leipert & Reutter, 2005; Mason & Agan, 2015; Masson et al., 2019; Mazorra et al., 2020; McCall et al., 2019; Ortega‐Egea et al., 2014; Patrick & Teresa, 2011; Powers, 2012; Roy & Venema, 2002; Seidel & Bell 2014; Shanthi et al., 2017; Shodieva et al., 2014; Singh et al., 2018; Tirado et al., 2013). Community‐based strategies to increase women empowerment were reported as mitigation strategies to address women's lack of access to education, health care and employment opportunities (Beaumier & Ford, 2010; Larson et al., 2018; Mason & Agan, 2015; Mazorra et al., 2020; Tirado et al., 2013). Strategies to enhance local adaptive capacity to climate change were also mentioned, with more input from women's perspectives regarding management at household levels (Drolet, 2012; Larson et al., 2018; Mason & Agan, 2015; Masson et al., 2019; Patrick & Teresa, 2011; Roy & Venema, 2002). Utilizing humanitarian resources to provide women with education around using renewable resources was noted as a solution to decreasing women's exposure to hazardous air pollutants during cooking times (Mazorra et al., 2020). Encouraging women to develop resilience, advocate for their rights, freedom of speech and equal involvement in decision making at a national level was also a reported mitigation strategy (Denton, 2002; Drolet, 2012; Granderson, 2017; Khapung, 2016; Koehler, 2016; Seidel & Bell 2014; Shanthi et al., 2017; Singh et al., 2018). Policy initiatives, taking into consideration the existing gender disparity, were highly recommended to improve societal conditions and women's access to health care services, especially maternal health care (Masson et al., 2019). Government assistance to women living in areas prone to extreme climatic effects, such as droughts, was found to mitigate health impacts of climate change on women in high‐income countries (HICs) (Powers, 2012). Women were noted to have higher resilience during times of distress, which was also reported as an adaptive strategy to address implications of climate change on women's health (Bunce et al., 2016; Leipert & Reutter, 2005; Masson et al., 2019; Powers, 2012).

## 4 Discussion

The scoping review has identified a strong but complex relationship between climate change and women's health. Most of the studies included in the review report findings from LMICs through qualitative study designs. The results identify robust evidence of the impact of climate change on women's health in LMICs, where currently most gender disparities exist (Bunce et al., 2016; Khapung, 2016; Powers, 2012). It is even more interesting to note that the small number of studies which were conducted in HICs were done so in rural and remote areas. This general finding indicates that gender inequality varies from rural to urban areas, but also highlights the need for more studies to analyze how women living in urban areas are affected by climate change.

Of the studies conducted in LMICs, it has been well established that climate change has influenced natural disasters and weather extremes that directly and indirectly affect women's health (Abdullah et al., 2019; Alhassan et al., 2019; Asamoah et al., 2018; Beaumier & Ford, 2010; Bunce et al., 2016; Cil & Cameron, 2017; Denton, 2002; Drolet, 2012; Khan et al., 2011; Leipert & Reutter, 2005; MacVicar et al., 2017; Marí‐Dell'Olmo et al., 2019; Mason & Agan, 2015; McCall et al., 2019; Poudel et al., 2020; Sanchez et al., 2012; Scheelbeek et al., 2016; Singh et al., 2018; Tirado et al., 2013; Zhang et al., 2018). Directly, women are more negatively affected by droughts and heat waves due to their roles in society and nutritional and physiological requirements during periods of menstruation and pregnancy (Beaumier & Ford, 2010; Denton, 2002; Koehler, 2016; Tirado et al., 2013). Women are already considered vulnerable populations globally due to societal conditions and the results from the scoping review indicate that this vulnerability also extends to the effects of climate change. Their role as being responsible for performing domestic housework duties and taking care of farmland at home, and be primary caregivers for children present a scenario where women are mostly homebound and unable to deal with the effects of natural disasters socially and physically. This indicates that there is potential for employment of community based educational programs to empower women in these settings to increase awareness of the climate change risks and adapt to reduce the health impacts.

The impact of climate change on maternal health has also been reported in the articles included in the scoping review. This relationship is very important because it is very closely related with pediatric health, and therefore overall population outcomes. Whilst pregnancy makes women physically vulnerable, immune system changes due to hormonal alterations are also sensitive to changes in temperatures. Heat stress impacts on both antibody and cell‐mediated immune responses (Nagai & Iriki, 2001), making them physiologically more vulnerable to acquiring infectious diseases, especially vector‐borne diseases, which has been well reported in the review (Denton, 2002; Bunce et al., 2016; Poudel et al., 2020). Birth outcomes and infant health are also affected due to the impact of climate change on maternal health, resulting in higher health care needs. It is reported that exposures to extreme temperatures during pregnancy can cause more birth defects in various climate zones around the world (Zhang et al., 2017). This is associated with negative health outcomes in the overall population in terms of utilizing already scarce medical resources and decreases sustainability of medical and health resources. This effect is likely to have a greater impact on population health in LMICs compared to HICs, where health care services and resources are more likely to be available. Mixed methods that incorporate both quantitative assessments (e.g., on increased maternal mortality and birth defects attributable to climate change) and qualitative studies (e.g., on socio‐cultural factors of gender inequity in the context of climate change) are strongly recommended to support evidence‐based policy making to protect women and children's health in changing climate.

The review has also identified factors that have been shown repeatedly, across various regions to render women consistently more vulnerable to climate change. Gender inequality is present in both HICs and in LMICs (Khapung, 2016; Powers, 2012). Women's lack of access to education, limited employment opportunities and minimal involvement in economic decision making further intensifies their vulnerability. If women do not have access to education and employment, they may not have access to information that may increase their awareness and understanding of climate change effects, which is an important enabling factor for adaptive changes at an individual and the societal level (Langer et al., 2015). This is especially important for women living in rural and remote areas where they already have limited access to resources and information. Globally, women predominantly face inequity in health care access due to societal and cultural factors (Masson et al., 2019; Mazorra et al., 2020). This calls for health care initiatives to identify and address these barriers as part of providing holistic health care for women to ensure that this gap is reduced in responding to climate change.

Adaptation and mitigation strategies have been discussed in majority of the included articles. Current societal conditions are identified as being the root cause of the vulnerability and negative heath impacts that women face (Alhassan et al., 2019; Beaumier & Ford, 2010; Masson et al., 2019; Roy & Venema, 2002). Strategies are outlined at an individual, community, national and global level in order to address the issue. At an individual level, building resilience to climate change effects is outlined as a strong approach that has the potential to underpin strategies at a national and global level (Leipert & Reutter, 2005; Mason & Agan, 2015). Due to their primary role as caretakers, women tend to care more about environmental change and adverse effects of climate change on future generation (Denton, 2002; Granderson, 2017; Mason & Agan, 2015; McCall et al., 2019; Ortega‐Egea et al., 2014). Building on this, it is beneficial to have insights from women in terms of adaptation strategies because they are more likely to provide perspectives on long‐term sustainable solutions. Women need to be empowered to participate in policy making process, especially when concerning use of natural resources such as energy and water. Community‐led strategies are also found to be effective in enabling and empowering poor rural women, for example, the “capabilities approach” to development and the “joint forestry management projects exclusively to rural women” in Indian (Roy & Venema, 2002). Moreover, having women only focus groups to share and express ideas and develop management strategies at the household level would be beneficial (Beaumier & Ford, 2010; Bunce et al., 2016).

In drawing attention to the global patterning of disadvantage confronting women as climate change advances, this review provides a powerful argument for the need for policy makers to have a gendered approach in decision making and acknowledge differing needs of women and men. HICs that have made progress in achieving this outcome need to share their knowledge and perspectives in helping reduce the gender inequality present in LMICs, where they are less likely to have resources to support women to achieve change. Our review indicates that complex interactions of social, cultural and economic factors that exist in today's society make climate change a gendered issue, by disproportionately impacting women's health. It is also noted that whilst adaptation and mitigation strategies were addressed in the studies, there is limited insights into barriers of implementing such policies and strategies, or assessment of community acceptance, feasibility of policies or cost implications.

There are a number of limitations present in the current scoping review. First, the scoping review excluded dissertations, theses, and books that may have provided further insights into the evidence in literature. Most studies have employed a qualitative study design which allowed insights into perspectives of different communities. However, there is always limitations in qualitative methods, such as subjectivity in analyzing qualitative data and difficulties in examining causality between climate change and health impacts. There is scope for more quantitative and mixed methods approaches to address different research questions for a better understanding of the extent of the problem, the vulnerability and health benefits of climate change responses. It will not only help fill in the gaps present in literature but also provide data to inform decision and policy making to allocate limited resources to the most vulnerable populations. The scoping review also did not assess the quality and strength of evidence presented in the articles included. Of the articles included, they were mainly based on data from LMICs, which may limit generalizability to HICs. This indicates that a gap in literature exists when assessing the impact of climate change on women's health in HICs.

## 5 Conclusion

The scoping review conducted indicates that women's health is at higher risks due to the vulnerability to climate change, especially in LMICs. These studies constituently suggested that societal, cultural, and economic factors are contributing to increased vulnerability of women. It is beneficial to have a gender aspect in climate change responses. Whilst most of the studies reported this relationship in the context of LMICs, it highlights the need for further research to be conducted in HICs' setting to allow a more comprehensive understanding of the scenario. Broadly, the themes of women's exposure to climate change risks, impacts on women's health, vulnerability and responding strategies are heavily underpinned by gender inequity issues. Recognizing these could assist implementation and increase effectiveness of climate change strategies from a societal perspective. Mixed methods are strongly recommended in future research to assist policy makers in responding to climate change. When considering development and implementation of climate change policies and strategies, it is important to acknowledge that the existing issue of gender inequity exacerbates the effects of climate change on women's health. Policies and strategies need to have a holistic approach and develop interventions according to different gender aspects.

## Conflict of Interest

The authors declare no conflicts of interests relevant to this study.

## Data Availability

This review paper did not analyze any new data. Only results published in identified previous studies were used. The 35 included studies were listed in Table 2 in the paper and in the reference list.
